# Characteristics of persistent arthritis with refractory Kawasaki disease: a single-center retrospective study

**DOI:** 10.1038/s41598-023-36308-9

**Published:** 2023-06-19

**Authors:** Seira Hattori, Tomo Nozawa, Kenichi Nishimura, Ryoki Hara, Ayako Murase, Asami Ohara, Ai Ohnishi, Takashi Ohya, Shuichi Ito

**Affiliations:** 1 Department of Paediatrics, Yokohama City University Graduate School of Medicine, 3-9 Fukuura, Kanazawa-Ku, Yokohama, Kanagawa 236-0004 Japan; 2 Department of Paediatrics, Nagasaki University Hospital, Nagasaki, Japan

**Keywords:** Rheumatic diseases, Musculoskeletal system, Rheumatic diseases

## Abstract

Arthritis is one complication of Kawasaki disease (KD); however, the clinical features of arthritis in KD have not been well clarified. We retrospectively investigated the characteristics of persistent arthritis beyond the subacute phase of KD. In this cohort, 49 of 243 patients (20%) developed arthritis, with 33 patients (14%) experiencing persistent arthritis. Among these 33 patients, 31 (94%) had complete KD. Thirty (91%) were resistant to first intravenous immunoglobulin, and 15 (45%) required additional infliximab. Five patients (15%) developed coronary artery lesions, and 24 (73%) had oligoarthritis, mainly in large lower-extremity joints. Twenty-four patients (73%) complained of arthralgia. At arthritis onset, 16 patients (48%) presented with fever, including recurrent fever in 10 patients. Serum C-reactive protein concentration in patients with active arthritis significantly increased compared with after acute KD treatment (2.4 vs. 0.7 mg/dL, *p* < 0.001). Serum matrix metalloproteinase-3, a biomarker of arthritis, was significantly higher in patients with active arthritis than in remission (93.7 vs. 20.3 ng/mL, *p* < 0.001). Thirty (91%) and 14 (42%) patients, respectively, were treated with non-steroidal anti-inflammatory drugs and prednisolone, and they completely recovered. To summarize, persistent arthritis is a common complication in refractory KD, and adequate diagnosis and treatment are necessary.

## Introduction

Kawasaki disease (KD) is an acute self-limiting vasculitis of unknown etiology that occurs in childhood^1^. The annual incidence of KD in Japan is increasing, with an incidence of 359 per 100,000 children aged 0–4 years^2^. The clinical course of untreated KD is generally divided into three phases: an acute febrile phase lasting 10–14 days; a subacute phase lasting 2–4 weeks with symptomatic improvement; and a convalescent phase whereby vessels undergo healing, remodeling, and scarring^3^. Coronary artery lesions (CALs) are the most common complications of KD. They develop during the subacute phase (or occasionally earlier) and can lead to myocardial ischemia and infarction^4^. Moreover, patients with KD present with various symptoms, including aseptic meningitis, anterior uveitis, hepatitis, gastroenteritis, gallbladder hydrops, pancreatitis, and arthritis^5^. Arthritis occurs in 4.6–17.6% of patients^6–11^; however, arthritis may be underdiagnosed as infants may struggle to express their symptoms. Arthritis associated with KD follows several clinical courses. Early-onset arthritis, which develops during the acute phase of KD (within 14th day of illness), is more frequently observed than late-onset arthritis, which develops primarily during the subacute phase or the convalescent phase^7^. Late-onset arthritis often persists for several weeks, and some patients require treatment. Although most cases of early-onset arthritis resolve with KD treatment during the acute phase, some patients experience persistent joint symptoms. Consequently, persistent arthritis may impact patients’ activities of daily living, and appropriate treatments, such as non-steroidal anti-inflammatory drugs (NSAIDs) or glucocorticoids, should be provided^6^. Novel diagnostic techniques, such as ultrasonography and magnetic resonance imaging (MRI), allow for easy observation of arthritis in patients with KD^9,12^, although detailed studies of persistent arthritis are lacking.

In the present study, we aimed to investigate the clinical features and courses of persistent arthritis defined as persisted to the subacute phase (i.e., after the 14th day of illness) or new-onset arthritis in the subacute phase in patients with KD. We also evaluated the efficacy of musculoskeletal ultrasonography (MSUS) and biomarkers of joint damage, such as matrix metalloproteinase-3 (MMP-3).

## Results

### Demographic and clinical features of patients with KD

A total of 243 patients were transferred to our institute, which is a regional center for intractable KD, during the study period because of resistance to intravenous immunoglobulin (IVIG) or other complications, including arthritis. The median age of the patients was 3.0 years (interquartile range [IQR], 1.8–5.0 years). Most of the patients were male (70%) and had complete KD (90.9%). As the primary treatment for KD, all patients received IVIG, although 225 patients (93%) were resistant to first IVIG treatment. Consequently, these patients were administered additional IVIG as a second-line therapy. As a third-line or subsequent therapy, 146 (60%), 28 (12%), 14 (5.8%), and 3 (1.2%) patients were treated with infliximab (IFX), plasma exchange, cyclosporine A (CyA), and methylprednisolone pulse therapy (MPT), respectively. Forty-four patients (18%) developed CALs, and 24 (9.9%) developed small aneurysms, 15 (6.2%) developed medium aneurysms, and five (2.1%) developed giant aneurysms.

**Figure 1:**
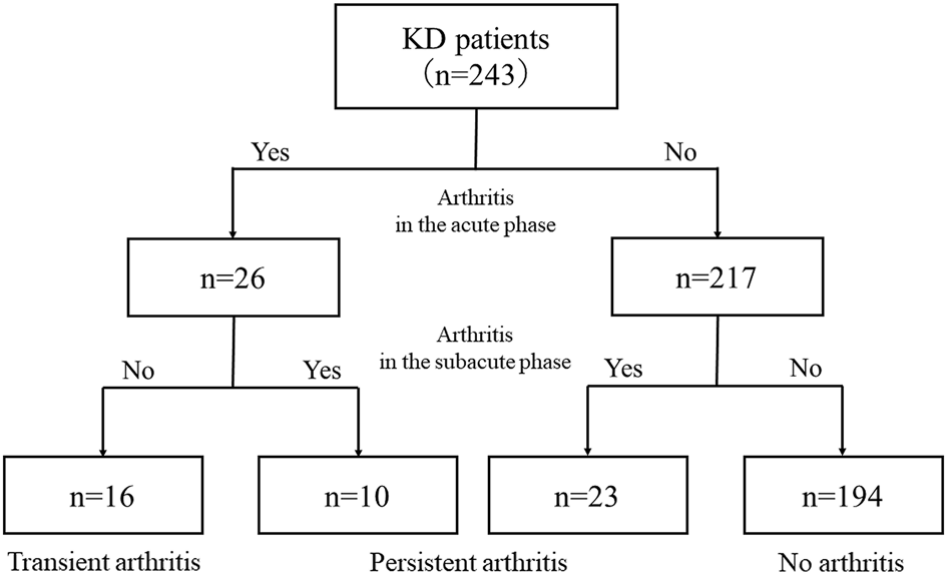
Patient flow in this study.

Of the 243 patients, 49 (20%) had arthritis at least once during KD (Fig. 1). The patients with arthritis were significantly older (median age: 3.8 vs. 2.8 years, *p* < 0.01) and had higher C-reactive protein (CRP) concentrations after acute treatment for KD (median: 1.08 vs. 0.50 mg/dL, *p* < 0.01) than those without arthritis, although there was no significant difference before treatment. There was no difference between the two groups in the past medical history of KD, family history of KD, CALs, or treatment, except for CyA. Patients with arthritis more often underwent treatment with CyA than those without arthritis (6 of 49 vs. 8 of 194, respectively, *p* = 0.041).

### Difference between early-onset and late-onset arthritis

Of the 49 patients with arthritis, 26 (53%) had early-onset arthritis and 23 (47%) had late-onset arthritis. Among the 26 patients with early-onset arthritis, 10 (38%) still had arthritis in the subacute phase of KD, and the other 16 recovered during the acute phase. Finally, 33 (14%) of 243 patients developed persistent arthritis in the subacute phase of KD.

Patients with early-onset arthritis were older (4.9 vs. 2.8 years, *p* = 0.030) and more often had polyarthritis (15 of 26 vs. 6 of 23, *p* = 0.042) affecting the elbows (5 of 26 vs. 0 of 23, *p* = 0.052) and fingers (13 of 26 vs. 4 of 23, *p* = 0.034), especially the proximal interphalangeal joint, than those with late-onset arthritis. There was no difference between the two groups in the past medical history of KD, family history of KD, CALs, or treatment, except for IFX. Patients with early-onset arthritis were more often treated with IFX than those with late-onset arthritis (22 of 26 vs. 9 of 23, respectively, *p* < 0.01).

### Clinical features of arthritis during the subacute phase of KD

The clinical features of persistent arthritis during the subacute phase of KD are shown in Table 1. Of 33 patients, 19 patients (58%) were male, and the median age was 3.7 years (IQR, 2.1–6.3 years). Seven patients (21%) had a past history of KD, and three patients (9.1%) had a family history of KD. Thirty-one patients (94%) had complete KD, while two patients (6.1%) had incomplete KD. Thirty patients (91%) were resistant to first IVIG, and 15 (45%), 6 (18%), 4 (12%), and 1 (3.0%) patient(s) were treated with IFX, plasma exchange, CyA, and MPT, respectively. Five patients (15%) had CALs.

**Table 1:** Clinical features of persistent arthritis during the subacute phase of KD.

	No. of patients (%), n = 33	
Clinical course of arthritis		
New onset during the subacute phase	23/33	(70)
Persistent from the acute phase	10/33	(30)
Arthralgia	24/30*	(80)
Type of arthritis		
Oligoarthritis (1–4 joints)	24/33	(73)
Polyarthritis (> 5 joints)	9/33	(27)
Affected joint		
Knee	25/33	(76)
Ankle	17/33	(52)
Hip	14/33	(42)
Wrist	9/33	(27)
Finger	8/33	(24)
Toe	6/33	(18)
Elbow	3/33	(9.1)
Neck	2/33	(6.1)
Shoulder	2/33	(6.1)
Fever	16/33	(48)
Persistent fever from the acute phase of KD	6/33	(18)
Recurrent fever with arthritis	10/33	(30)

*The other three patients were infants.

*IQR* interquartile range, *KD* Kawasaki disease.

Of the 33 patients with persistent arthritis, 23 (70%) had new-onset arthritis during the subacute phase, and 10 (30%) had persistent arthritis from the acute phase. The median diagnosis date of new-onset arthritis during the subacute phase was the 20th day of illness (IQR, 16–24 days). Twenty-four patients (80%), excluding three infants, experienced arthralgia. Twenty-four patients (73%) had oligoarthritis, mainly involving the large joints. The predominantly affected joints were the knees (76%), ankles (52%), and hips (42%). In total, large weight-bearing joints in the lower extremities were affected in 32 patients (97%). Sixteen patients (48%) had fever associated with arthritis. Among them, 6 patients (18%) had prolonged fever from the acute phase of KD, and 10 (30%) had recurrent fever from arthritis onset.

### Laboratory examinations (CRP and MMP-3) and MSUS in patients with persistent arthritis

**Figure 2:**
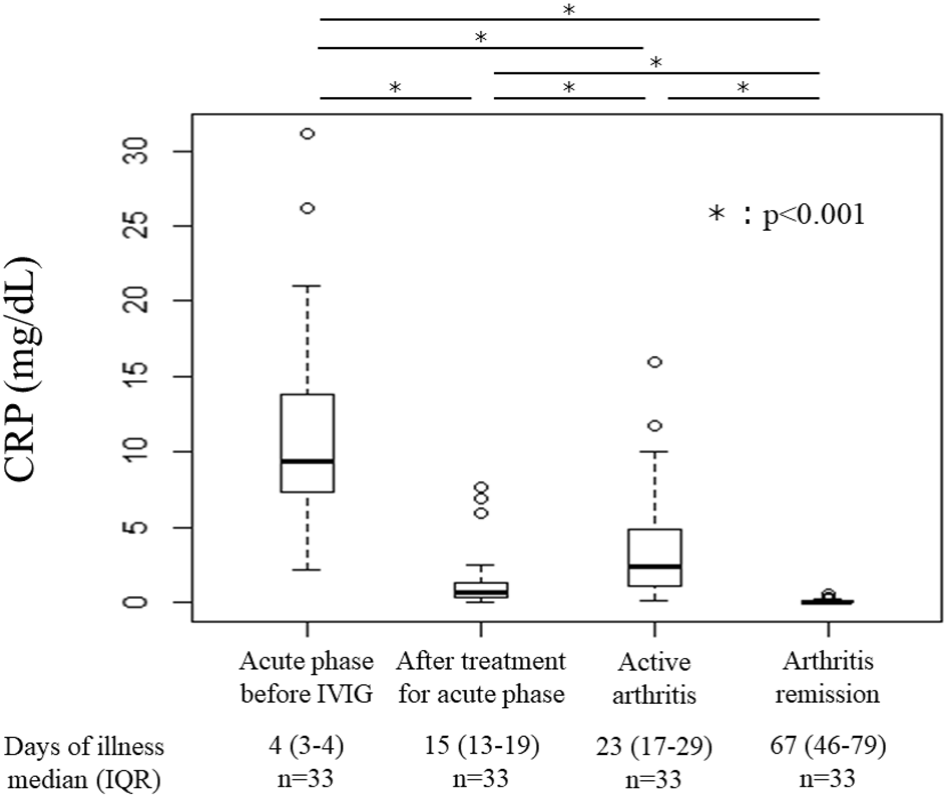
Time-dependent changes in serum C-reactive protein (CRP) concentration in patients with persistent arthritis.

The time-dependent changes in serum CRP concentration are shown in Fig. 2. The median serum concentration of CRP during the acute phase before IVIG, after treatment for the acute phase of KD, during active arthritis, and during arthritis remission was 9.3 mg/dL (IQR, 7.3–13.8 mg/dL), 0.7 mg/dL (IQR, 0.3–1.4 mg/dL), 2.4 mg/dL (IQR, 1.1–4.8 mg/dL), and 0.03 mg/dL (IQR, 0.02–0.10 mg/dL), respectively. The serum CRP concentration during the active phase of arthritis was significantly higher than after treatment for KD during the acute phase (*p* < 0.001). Re-elevation of the CRP concentration during the subacute phase of KD was observed in 30 patients (91%), most cases of which were observed after the reduction in the acetylsalicylic acid (ASA) dose to 5 mg/kg/day.

**Figure 3:**
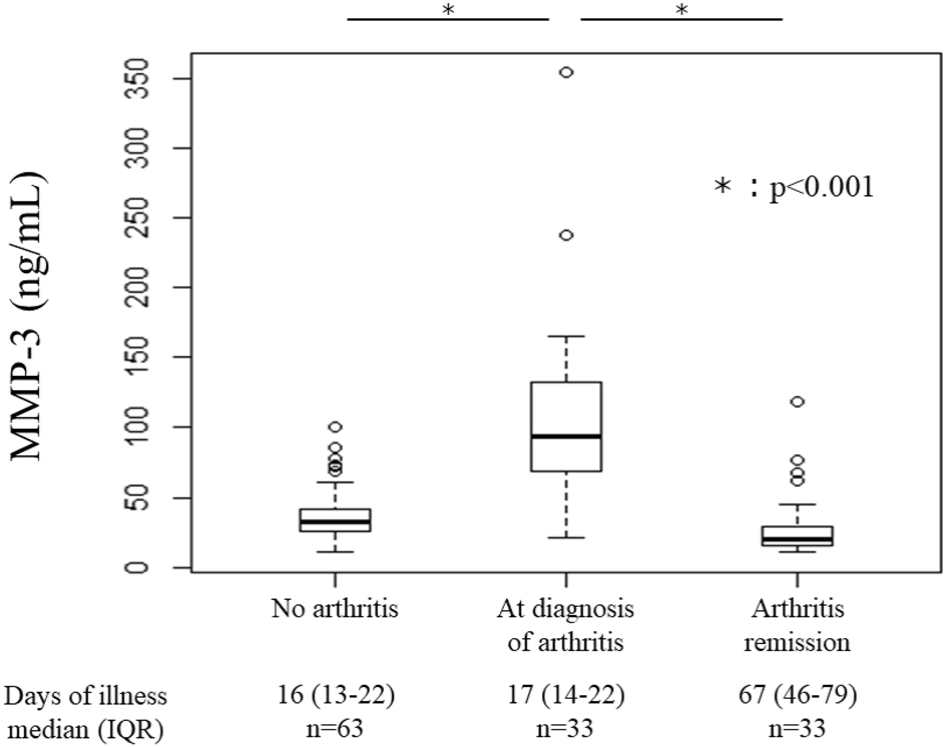
Serum matrix metalloproteinase-3 (MMP-3) concentration in the active and remission phases of arthritis.

The median serum concentration of MMP-3 at arthritis diagnosis and during arthritis remission was 93.7 ng/mL (IQR, 68.9–132.3 ng/mL) and 20.3 ng/mL (IQR, 15.0–28.2 ng/mL, *p* < 0.001), respectively (Fig. 3). The serum MMP-3 concentration during the active phase of arthritis was > 15.0 ng/mL in all patients. Among the 194 patients without arthritis, 63 underwent measurement of serum MMP-3 during the subacute phase of KD, and the median serum MMP-3 concentration was 33.9 ng/mL (IQR, 27.8–44.3 ng/mL), which was significantly lower than the serum MMP-3 concentration of patients with persistent arthritis (*p* < 0.001).

**Figure 4:**
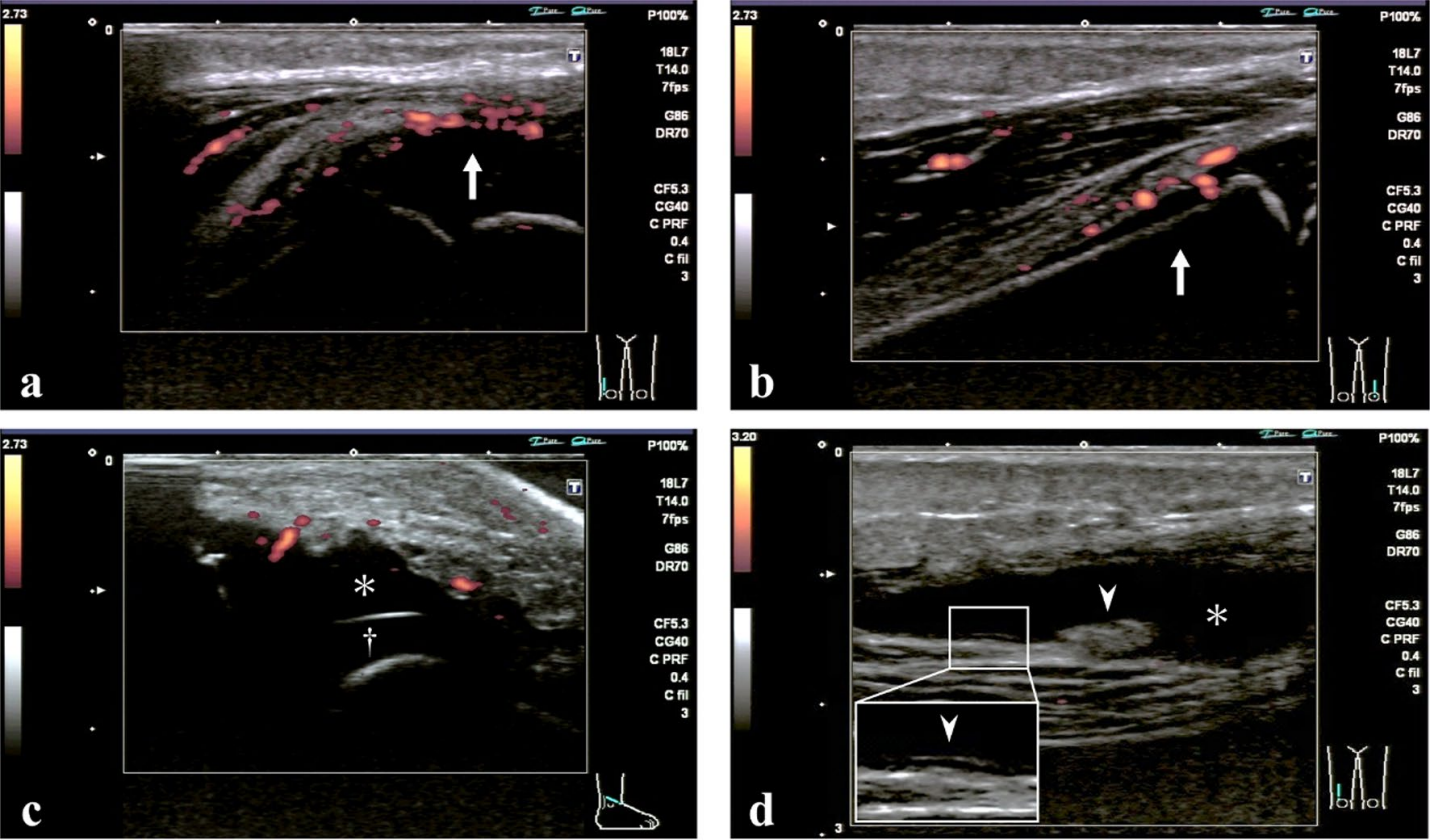
Representative musculoskeletal ultrasonography images. (**a**, **b**) Images from a boy aged 2 years. The knees were examined bilaterally revealing PD signals (arrow) on the periarticular soft tissue. (**c**) Image from a boy aged 1 year. Increased joint fluid (*) was observed on the cartilage (†) in the right ankle. (**d**) Image from a boy aged 7 years. Increased joint fluid (*) was observed in the right knee, as was linear/nodular edematous thickening of the synovium (arrowhead).

Thirty patients underwent MSUS during the active phase of arthritis. Positive power Doppler (PD) signals on periarticular soft tissue, fasciae, tendons, and fat were observed in 28 patients (93%), and joint fluid was observed in 27 patients (90%). However, no patients showed PD signals on synovial proliferation, although 16 patients (53%) showed linear or nodular edematous thickening of the synovium without PD signals (Fig. 4). Synovial thickening, joint fluid, and PD signals on periarticular soft tissue disappeared within several weeks.

### Treatment for arthritis and outcomes

The following NSAIDs were administered among 30 patients (91%) for arthritis: naproxen (n = 18), high-dose ASA (n = 8; 50 mg/kg/day) for anti-inflammatory effects, ibuprofen (n = 3), and flurbiprofen (n = 1). Among the patients administered high-dose ASA, two were changed to naproxen and one to ibuprofen because the dose of ASA was reduced (5 mg/kg/day) for antiplatelet effects. The median duration of treatment with NSAIDs was 35 days (IQR, 20–47 days).

Prednisolone (PSL) was administered in 14 patients (42%) because of NSAIDs resistance. PSL was initiated at 0.5–1.5 mg/kg/day and gradually tapered. The median time from onset to PSL initiation was 28 days (IQR, 25–33 days). Treatment with PSL improved arthritis quickly in all patients. The median duration of treatment with PSL was 56 days (IQR, 43–88 days). Two patients (6.1%) did not require treatment for arthritis and achieved spontaneous remission within 1 month. No patients received biological agents for the treatment of arthritis. All patients achieved remission of arthritis within 3 months. At the final observation, no patients had joint symptoms and sequelae, so the arthritis prognosis was good. There were no severe adverse events in response to treatment, and the number of patients with CALs did not increase.

## Discussion

In this study, we demonstrate that persistent arthritis is a common complication in patients with refractory KD. Persistent oligoarthritis frequently occurs in large weight-bearing joints in the lower extremities. Recurrent fever and re-elevation of the serum CRP concentration could reflect active arthritis. Serum MMP-3 concentration and MSUS were helpful for the diagnosis of arthritis. Moreover, the response to treatment, including NSAIDs and PSL, and the joint prognosis were favorable.

Arthritis is a classic complication of KD that occurs in 4.6–17.6% of patients^6–11^, but it has been improperly evaluated and underdiagnosed for decades. Infants and toddlers may have difficulty complaining about their joint symptoms, which could contribute to the underdiagnosis. For physicians, it is difficult to notice swelling in the small joints of infants. However, recently, arthritis in KD has garnered substantial attention^7–11^. Oligoarthritis accounts for 56.0–82.5% of cases of KD-associated arthritis^6–10^, and large joints in the lower extremities are more frequently involved than small joints^6,8,9^. Our results are consistent with those of previous studies ^6^ showing that oligoarthritis was predominantly observed (64%) and large joints in the lower extremities were primarily affected. Arthritis persisted for several weeks in 10 patients (38%) from the acute phase of KD. However, no prior studies have examined whether arthritis that develops in the acute phase of KD can persist through the subacute phase. Therefore, certain patients who are diagnosed with late-onset arthritis may be underdiagnosed during the acute phase. Most cases of early-onset arthritis resolve quickly following initiation of IVIG and high-dose ASA^6,8,9^. However, late-onset arthritis often requires additional treatment^7,10,13^, although there are only a few reports on the treatment of arthritis at this stage. Regardless of the timing of onset, the long-term prognosis of KD-associated arthritis is good^6–10,13^. However, in our study, 24 patients (80%), excluding three infants, complained of arthralgia, and arthralgia and fever are potential reasons for therapeutic intervention. NSAIDs were used in 30 patients (91%) in the present study, although 14 patients (42%) required short-term treatment with PSL. The response to glucocorticoids was rapid and highly satisfactory in most patients, and PSL was successfully tapered and discontinued within a few months. Regardless of the time of onset, persistent arthritis can affect the quality of life of patients and often requires treatment. In our study, five patients (15%) developed coronary artery aneurysm. Whether patients with concomitant arthritis are more likely to develop coronary artery aneurysm is controversial^7,8,10^.

According to the biennial nationwide survey of KD in Japan, patients with complete KD and IVIG resistance account for 78.9% and 19.7% of all patients, respectively^2^. In our study, patients with complete KD and IVIG resistance accounted for 94% and 91% of patients, respectively. Patients with KD who are resistant to IVIG are reported to develop persistent arthritis^7,13^. Furthermore, three (9.1%) and seven (21%) patients in our study had a family history of KD and a past history of KD, respectively. In the Japanese nationwide survey, family history was observed with siblings and parents in 2.2% and 1.3% of patients, respectively, and KD recurrence was observed in 4.6% of the patient population^2,14^. These findings may suggest that high susceptibility to KD and KD severity are risk factors for persistent arthritis.

During the active phase of arthritis, among the physical symptoms, fever is frequently observed. In this study, six patients (18%) experienced persistent fever from the acute phase of KD, and 10 patients (30%) developed recurrent fever with arthritis onset. According to previous reports, recurrent and persistent fever may occur in patients with late-onset arthritis^7,13^. Meanwhile, recurrent and persistent fever can be recognized as symptoms of KD flare-ups. Recurrent fever and smoldering symptoms are reported as risk factors for coronary aneurysm^15,16^. For proper treatment, arthritis-associated fever and KD-associated systemic vasculitis must be differentiated. Notably, serum MMP-3 was significantly elevated during the acute phase of arthritis in all patients. Serum MMP-3 is a biomarker of joint cartilage injury, reflecting disease activity in rheumatoid arthritis and juvenile idiopathic arthritis (JIA)^17^. Although glucocorticoids are reported to increase the MMP-3 concentration^18^, in our study, only one patient was administered glucocorticoids before arthritis onset. The normal MMP-3 concentration range in children is unclear, although Matsuyama et al. showed that the median MMP-3 concentration in healthy children is 12.0 ng/mL (range, 5–18 ng/mL)^19^, and Japanese clinical practice guidelines for JIA indicate a lower concentration compared with adults (< 15.0 ng/mL)^20^.

Regarding MSUS evaluation, joint fluid was observed in most patients, although none had synovial proliferation accompanied by PD signals, which is commonly observed in the articular form of JIA. These observations are consistent with a previous report^9^. Additionally, some patients showed edematous thickening of the synovium without PD signals, which resolved quickly as arthritis improved. Positive PD signals on the periarticular soft tissue, fasciae, tendons, and fat were observed in almost all patients, and these findings disappeared as arthritis improved, which may explain the pathogenesis and favorable prognosis of arthritis in KD. According to a previous case report, gadolinium-enhanced MRI in a patient with KD showed contrast effects on thigh muscles, suggesting myositis^12^. In another report, a biopsy of the quadriceps muscle in a patient with KD showed vasculitis^21^. KD-associated vasculitis may affect numerous musculoskeletal systems. In a previous study, arthrotomy of the hip in a patient with KD revealed turbid joint fluid with white blood cells, but no synovial hypertrophy^22^. These findings suggest that KD-associated arthritis may not be primary synovitis, such as JIA; rather, it may be secondary arthritis complicated with systemic vasculitis involving the musculoskeletal system. These differences between KD and JIA may partially explain why IFX is ineffective for KD-associated arthritis. In our study, over half of patients received IFX (n = 15 [45%]) as a third-line or subsequent therapy. It is interesting to note that arthritis occurred in patients who underwent IFX treatment. IFX, which is a monoclonal anti-tumor necrosis factor (TNF)-α antibody, is highly effective for treating JIA^23^. Therefore, TNF-α may not play a central role in KD-associated arthritis. Furthermore, in our region, only a very limited number of hospitals administer IVIG plus PSL as initial treatment for KD based on a predicted score for IVIG failure^24,25^. This allowed us to observe the natural history of arthritis because the effects of PSL could be ignored.

This study has several limitations that should be noted. First, there was bias in the background of the study population. The population consisted of patients with severe KD because our hospital is a regional center for intractable KD. Therefore, we could not estimate the incidence of arthritis or the ratio of early-onset to late-onset arthritis among all patients, but the incidence of arthritis in IVIG-resistant patients was likely accurate. Second, it was difficult to recognize arthritis in patients without fever, or re-elevation of CRP, especially in infants. Consequently, arthritis may have been underdiagnosed in these patients.

In conclusion, patients with KD who suffer from persistent arthritis tend to have fever and re-elevation of the CRP concentration. Evaluation of the MMP-3 concentration and MSUS findings, particularly enhanced PD signals on periarticular soft tissue, joint fluid, and edematous thickening of synovium without PD signals, may help to diagnose arthritis in patients with poor findings.

## Methods

### Patients and study design

This was a single-center retrospective study of patients with KD treated at Yokohama City University Hospital between April 2008 and March 2019. We investigated KD with or without arthritis, whether it was early or late-onset, and persistent arthritis in the subacute phase of KD. We defined persistent arthritis as arthritis that persisted to the subacute phase (i.e., after the 14th day of illness) or new-onset arthritis in the subacute phase, while transient arthritis as arthritis that improved in the acute phase. Despite our institute being a regional KD center, we mostly encounter intractable patients with IVIG resistance or severe complications, such as CALs. We defined refractory KD as persisting systemic symptoms or complications, such as arthritis, despite the first IVIG treatment. The first-line therapy for KD at other regional collaborating hospitals is IVIG (2 g/kg/dose) with ASA (30 mg/kg/day), while second-line therapy involves additional IVIG (2 g/kg/dose). PSL is not used in our region, even when the risk score for predicting IVIG resistance is high^24^. Patients were diagnosed with KD, including incomplete KD, according to Japanese diagnostic guidelines^26^. Additionally, arthritis was diagnosed based on physical examinations^23^ or ultrasonography and MRI, which allow for visualization of joint fluid, if necessary. CALs were defined according to Z-scores as follows: dilation: 2 to < 2.5; small aneurysm: ≥ 2.5 to < 5; medium aneurysm: ≥ 5 to < 10 and an absolute dimension of < 8 mm; large or giant aneurysm: ≥ 10 or an absolute dimension of ≥ 8 mm^6,27^. Oligoarthritis was defined as arthritis with fewer than five affected joints, while polyarthritis was defined as arthritis with five or more affected joints.

### Data collection

The following data were retrospectively collected through a review of electronic medical records in all patients: sex, age at diagnosis of KD, type of KD, history of KD, family history of KD, treatment for the acute phase of KD, IVIG resistance, presence of CALs, and serum concentration of CRP before and after treatment for the acute phase. Furthermore, in patients with arthritis, we investigated the number of days from KD onset to arthritis onset, the number of affected joints, the presence of fever and arthralgia in the subacute phase of KD, the serum concentrations of CRP and MMP-3 in active arthritis and remission, the findings from MSUS, arthritis treatment, and joint sequelae.

### Statistical analysis

Continuous variables are presented as median with either range or IQR, as indicated, and categorical variables are presented as frequency and percentage. The Mann–Whitney U test was performed to compare the CRP and MMP-3 concentrations between patients with KD with or without arthritis. Friedman’s two-way analysis of variance by ranks was performed to compare the CRP concentrations of each phase in patients with KD with persistent arthritis. Wilcoxon’s signed-rank test with Bonferroni correction was performed as a post-hoc test when significant differences were observed. Wilcoxon’s signed-rank test was performed to compare the MMP-3 concentration between the active and remission phases of arthritis. Fisher’s exact test was performed to compare categorical variables between two groups. All analyses were performed using R statistical package, version 3.5.3^28^, and statistical significance was set at *p* < 0.05.

### Ethics

This study was in compliance with the Declaration of Helsinki and approved by the ethics committee of Yokohama City University Hospital (No. B200600052). Informed consent was waived, as this study was a retrospective and non-invasive study using data from electronic medical charts and the patient’s parents did not opt-out of their data being used for this study.

## Data Availability

The data of this study are available from the corresponding author on reasonable request.
